# Ideotype based genotype selection in a multivariate dataset of sweet potato (*Ipomoea batatas* L.)

**DOI:** 10.1016/j.dib.2024.110575

**Published:** 2024-06-01

**Authors:** Zakaria Alam, Sanjida Akter, Md. Anwar Hossain Khan, Atiqur Rahman, Md Hasan Sofiur Rahman

**Affiliations:** a Tuber Crops Research Centre, Bangladesh Agricultural Research Institute, Gazipur 1701, Bangladesh; b Entomology Division, Bangladesh Rice research Institute, Bangladesh; c Bangladesh Agricultural University, Mymensingh, Bangladesh; d Plant Breeding Division, Bangladesh Institute of Nuclear Agriculture, Mymensingh, Bangladesh

**Keywords:** Correlation, Principal component analysis (PCA), Multi trait genotype-ideotype index (MGIDI), Factor analysis, Genetic gain, Broad sense heritability

## Abstract

The dataset extensively examines the factors considered when choosing sweet potato genotypes, considering various characteristics. Notably, Moz1.15 demonstrated the highest marketable root yield at 46.46 t/ha, H5.ej.10 exhibited the highest beta-carotene level at 48.94 mg/100 g, and Moz1.9 recorded the highest vitamin C content at 23.89 mg/100 g. Moreover, there were significant correlations (ranging from 0.21 to 0.84) among the yield and quality traits studied in sweet potatoes. Principal component analysis (PCA) confirmed the connections among these traits, identifying four distinct clusters of genotypes, each characterized by specific significant combinations of traits. Factor analysis using the multi-trait genotype-ideotype index (MGIDI) highlighted the considerable impact of sweet potato traits across two growing seasons (2020–21 and 2021–22), facilitating the selection of genotypes with potential genetic gains ranging from 1.86 % to 75.4 %. Broad-sense heritability (h^2^) varied from 64.9 % to 99.8 %. The use of the MGIDI index pinpointed several promising genotypes, with BARI Mistialu-12 and H9.7.12 consistently performing well over both years. These genotypes exhibited both strengths and weaknesses.

Specifications Table

**Table:** 

Subject	Agricultural and Biological Science
Specific subject area	Agronomy and Crop Science
Data format	Raw
Type of data	Table and Figures
How the data were collected	Harvesting of sweet potato roots was done at 130 days after planting of sweet potato vines/cuttings, with a random selection of 10 plants from each plot for every replication. Using slide callipers, various measurements were taken, and the analytical balance was employed for weighing. Storage roots weighing 500 g from each genotype per replication were collected, and quality data was analyzed in the laboratory.
Data source location	The trial was carried out over two successive growing periods (2020–21 and 2021–22) in Bogura, Bangladesh. The research site is positioned at a latitude of 23.6850° N and a longitude of 90.3563° E, with an elevation of 10 m above sea level in the Coastal South and 105 m above sea level in the North.
Data accessibility	https://data.mendeley.com/datasets/n8cncsmrym/1
Related research article	Z. Alam, S. Akter, M. A. H. Khan, M. N. Amin, M. R. Karim, M. H. S. Rahman, M. H. Rashid, M. M. Rahman, N. Mokarroma, A. A. Sabuz, M. J. Alam, Multivariate analysis of yield and quality traits in sweet potato genotypes (Ipomoea batatas L.), Scientia Horticulturae, 328 (2024) p.112901. https://doi.org/10.1016/j.scienta.2024.112901

## 1 Value of the Data

• The dataset delves into intricate trait interplays influencing sweet potato production and quality, spotlighting the adaptable performances of various genotypes across traits.
• It emphasizes the importance of improving both yield and quality, essential for sweet potato stakeholders including farmers and the industry.
• By employing the MGIDI index, distinct high-quality genotypes are pinpointed, providing breeders with valuable insights into their pros and cons. This methodology establishes a cornerstone for upcoming research and breeding endeavors, tackling global food and nutritional security issues.

## 2 Background

The government of Bangladesh is addressing challenges related to water scarcity and the overreliance on paddy cultivation by promoting the cultivation of alternative crops on unused lands [1]. In the northern riverine regions, where sandy soils and water scarcity complicate rice and cereal farming, there is a shift towards sweet potatoes. Cultivated near rivers and riverine islands, sweet potatoes not only provide a solution to water scarcity but also offer a rich source of beta-carotene, vitamin C, flavonoids, and phenolic acids, with a low glycemic index, contributing to improved nutritional outcomes [2]. The success of sweet potato production depends on various factors such as genotypes and environments, as emphasized in recent research [[3], [4], [5]]. To address these complexities, the adoption of the plant ideotype concept in selecting genotypes has emerged as a suitable solution. The dataset accompanying this information includes four tables and three figures.

## 3 Data Description

### 3.1 Genotypic performance and variance analysis of sweet potato

Table 1 presents an ANOVA for various yield and quality parameters in sweet potato genotypes. The results show that several parameters, such as marketable root yield (MRY), marketable root number (MRN),average root diameter (ARD), vitamin C (VC), beta-carotene (BC), storage root weight (ST), total soluble solids (TS), average root circumference (ACD), dry weight (DW), and total soluble solids (TSS), were statistically significant at a significance level of *p*≤0.05 across different genotypes. Particularly noteworthy are the significant differences in BC, VC, TS, and DW observed between the two study years (2020–21 and 2021–22).

**Table 1:** Combined ANOVA of yield and quality traits of 17 sweet potato genotypes grown in Bogura, Bangladesh during two consecutive growing seasons (2020–21 and 2021–22).

Source of variation	df	Mean sum of squares													
		VL	ARL	ARD	MRN	NMRN	NMRW	MRY	BC	VC	ST	TS	ACD	DW	TSS
Years	1	350.79	0.14	6.12	16.34	0.002	0.002	277.37	8.957*	11.023*	2.52	2.4941*	0.00003	18.48**	3.58
Replication within Years	4	70.46	2.25	9.20***	4.78***	2.34	0.0006	39.03	0.78	1.39	1.38	0.2828	0.0017	0.68	2.46
Genotypes	16	1547.43***	4.40**	1.03	4.71***	0.28	0.0002	242.33***	726.67***	149.60***	1.64*	13.372***	0.0299***	153.43***	13.26***
Genotypes × Years	16	62.04	1.58	0.24	0.79	0.39	0.0001	38.10	0.6	0.41	1.02	0.149	0.0001	1.39	0.19
Pooled Error	64	98.55	1.61	0.79	0.62	0.43	0.0002	28.54	1.2	0.52	0.76	0.282	0.0002	0.96	0.298
Total	101														

df= degrees of freedom, VL=vine length, ARL=average storage root length, ARD=average storage root diameter, MRN=marketable storage root number per plant, NMRN=average non-marketable storage root number per plant, NMRW=average non-marketable storage root weight per plant, MRY= Marketable storage root yield, BC=Beta-carotene content, VC= Vitamin-C, ST= starch, TS= total sugar, ACD= titratable acidity, DW=dry weight of storage root, TSS= total soluble solid, *** significant at 0.001% level of probability, ** significant at 0.01% level of probability, * significant at 0.05% level of probability, ns=non-significant

Table 2 presents the average values of various traits across different genotypes. Regarding traits related to yield, BARI Mistialu-12 showed the longest vine length (VL) at 149.17 cm. H9.10.12 had the highest average root length (ARL) at 15.34 cm. Moz1.15 exhibited the highest marketable root number (MRN) at 7.17 kg. This genotype also achieved the highest marketable root yield (MRY) at 45.46 t/ha among all sweet potato genotypes. Concerning quality traits, BARI Mistialu-12 had the highest dry weight (DW) at 36.11 %. H5.ej.10 displayed the highest beta-carotene (BC) content at 48.94 mg/100 g. Moz1.9 recorded the highest vitamin C (VC) content at 23.8 mg/100 g. H6.52.11 exhibited the highest storage root weight (ST) at 42.01 %. The highest total soluble solids (TSS) content was observed in Moz1.9 at 12.26 %. Average root circumference (ACD) values varied, with BARI Mistialu-15 having the highest at 0.33 %. Moz1.9 also had the highest total soluble solids (TSS) content at 12.86 %.

**Table 2:** Mean values of 17 sweet potato genotypes for yield and quality traits studied in Bogura in two consecutive growing years.

ID	Genotypes	VL (cm)	ARL (cm)	MRN	MRY (t/ha)	DW (%)	BC (mg/100g)	VC (mg/100g)	ST (%)	TS (%)	ACD (%)	TSS (Brix%)
1	Moz1.15	109.13^ef^	13.88^a-e^	7.17^a^	45.46^a^	23.45^fg^	8.87^g^	5.35^de^	41.21^a-d^	11.23^bcd^	0.13^e^	11.00^cd^
2	Moz1.9	143.93^ab^	15.14^ab^	5.19^efg^	32.60^de^	33.54^b^	8.97^g^	23.89^a^	41.84^ab^	12.26^a^	0.31^b^	12.86^a^
3	SPM103	106.38^f^	14.54^abc^	5.31^def^	30.45^def^	20.65^i^	9.48^g^	11.36^b^	41.07^a-d^	10.50^fg^	0.13^e^	9.51^ef^
4	SPO104	111.17^ef^	13.95^a-e^	5.35^c-f^	32.78^de^	21.95^h^	13.07^f^	5.04^def^	40.26^d^	9.61^hi^	0.13^e^	9.31^ef^
5	H9.7.12	136.23^bc^	14.20^a-d^	6.71^ab^	42.04^ab^	31.90^c^	13.88^f^	9.16^c^	40.52^cd^	9.36^i^	0.13^e^	9.30^ef^
6	H9.10.12	104.72^f^	15.34^a^	6.43^ab^	39.62^abc^	19.89^i^	21.51^c^	5.07^def^	40.61^cd^	9.09^i^	0.13^e^	9.01^fg^
7	H6.52.11	106.50^f^	14.80^ab^	6.03^b-e^	35.58^cd^	22.84^gh^	13.30^f^	5.41^d^	42.01^a^	11.18^b-e^	0.20^c^	11.05^cd^
8	H9.48.11	106.17^f^	14.56^abc^	6.25^bc^	42.25^ab^	22.53^gh^	6.66^h^	5.40^d^	40.79^cd^	11.75^ab^	0.21^c^	10.64^d^
9	H5.ej.10	105.00^f^	14.61^abc^	5.04^fg^	31.02^def^	23.34^fg^	48.94^a^	4.92^def^	40.66^cd^	10.05^gh^	0.13^e^	9.71^e^
10	H16.ej.10	133.92^bc^	13.85^b-e^	4.90^fg^	28.94^ef^	28.53^d^	10.01^g^	4.50^fg^	40.38^d^	10.59^efg^	0.13^e^	10.98^cd^
11	BARI Mistialu-12	149.17^a^	15.11^ab^	6.86^ab^	42.43^ab^	36.10^a^	17.38^e^	4.54^efg^	40.93^bcd^	10.54^fg^	0.14^e^	10.91^cd^
12	BARI Mistialu-16	104.00^f^	14.42^a-d^	5.38^c-f^	36.52^bcd^	19.60^i^	21.44^c^	4.40^fg^	40.76^cd^	5.88^j^	0.14^e^	6.57^h^
13	BARI Mistialu-17	113.83^ef^	13.92^a-e^	6.10^bcd^	33.86^cde^	26.23^e^	16.71^e^	11.96^b^	40.47^cd^	10.65^d-g^	0.22^c^	8.58^g^
14	BARI Mistialu-8	128.03^cd^	12.51^e^	4.29^g^	25.33^f^	29.11^d^	34.92^b^	3.80^g^	41.48^abc^	11.37^bc^	0.08^f^	11.41^c^
15	BARI Mistialu-13	119.67^de^	13.08^de^	4.87^fg^	26.17^f^	26.76^e^	19.26^d^	4.94^def^	40.78^cd^	10.99^c-f^	0.18^d^	10.70^d^
16	BARI Mistialu-14	119.93^de^	12.53^e^	4.38^g^	26.11^f^	24.08^f^	17.21^e^	5.38^d^	40.32^d^	9.45^hi^	0.08^f^	9.49^ef^
17	BARI Mistialu-15	145.97^ab^	13.31^cde^	4.68^fg^	29.37^ef^	32.01^c^	3.97^i^	3.92^g^	41.42^abc^	12.08^a^	0.33^a^	12.15^b^
Mean		120.22	14.10	5.58	34.15	26.03	16.80	7.00	40.91	10.39	0.16	10.19
CV (%)		8.25	9.01	14.04	15.64	3.76	6.52	10.30	2.13	5.11	9.08	5.36
LSD_0.05_		12.15	1.47	0.90	6.16	1.13	1.26	0.83	1.00	0.61	0.02	0.63

CV= co-efficient of variations, LSD_0.05_ = least significant difference at 5% probability level, VL=vine length, ARL=average storage root length, ARD=average storage root diameter, MRN=marketable storage root number per plant, NMRN=average non-marketable storage root number per plant, NMRW=average non-marketable storage root weight per plant, MRY= Marketable storage root yield, BC=Beta-carotene content, VC= Vitamin-C, ST= starch, TS= total sugar, ACD= titratable acidity, DW=dry weight of storage root, TSS= total soluble solid

In reference to the different growing seasons (Table 3), the sweet potato genotype cultivated in the 2021–22 period displayed the highest dry weight (DW) at 26.46 %. The peak beta-carotene (BC) content was observed during the 2021–22 season, reaching 17.10 mg/100 g. In the same season, vitamin C (VC) reached its highest value at 7.33 mg/100 g. The highest total soluble solids (TS) content was observed during the 2021–22 season, reaching 10.54 %.

**Table 3:** Mean values of two growing years for quality traits of 17 sweet potato genotypes studied in Bogura, Bangladesh.

Years	DW	BC	VC	TS
2020–21	25.60^b^	16.50^b^	6.67^b^	10.23^b^
2021–22	26.46^a^	17.10^a^	7.33^a^	10.54^a^
CV (%)	3.16	5.26	16.85	5.12
LSD (0.05)	0.45	0.49	0.65	0.29

CV= co-efficient of variations, LSD (0.05) = least significant difference at 5% probability level, DW=dry weight of storage root, BC=Beta-carotene content, VC= Vitamin-C, TS= total sugar

### 3.2 Co-efficient of correlations among sweet potato traits

**Fig. 1:**
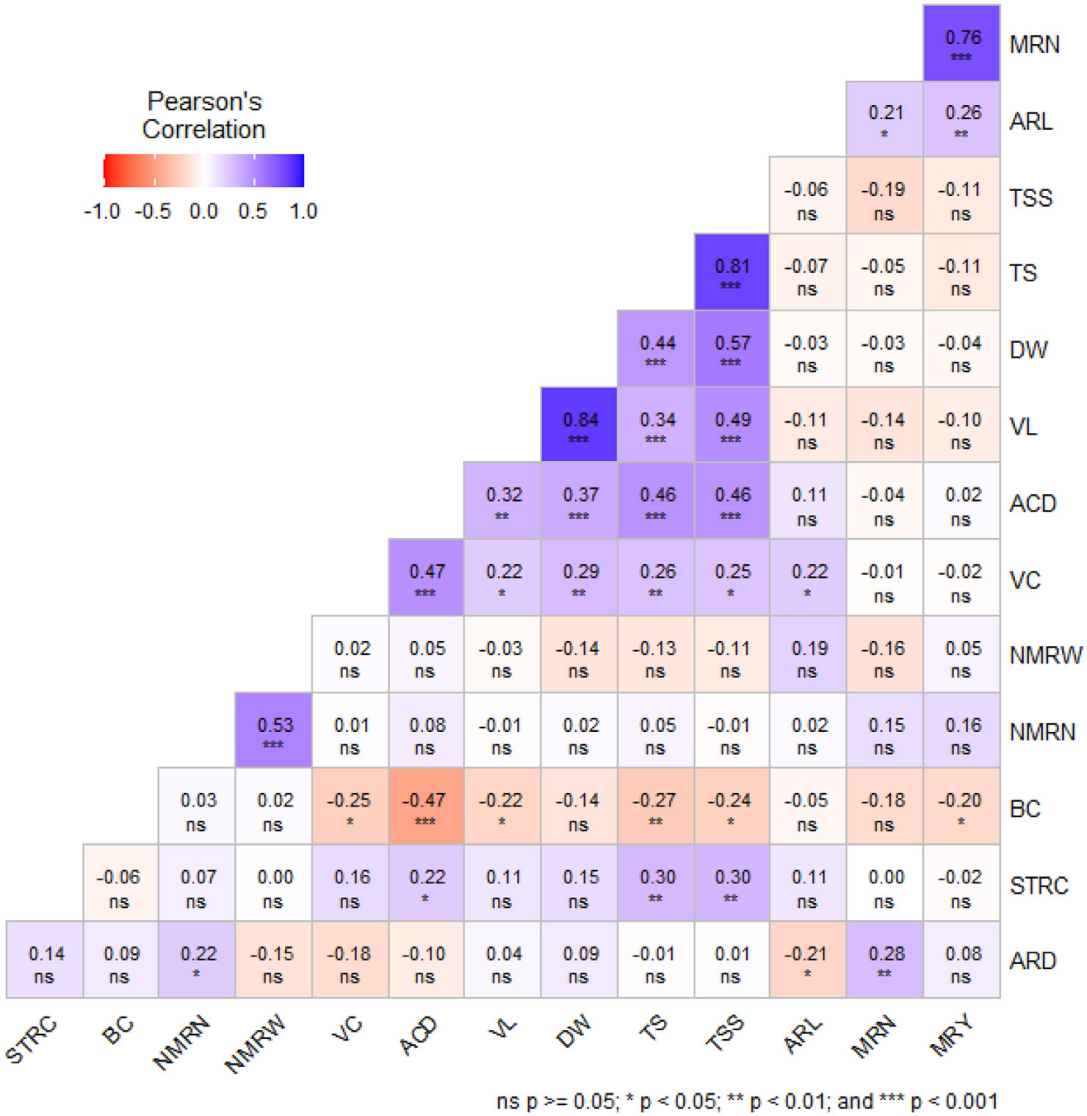
Linear correlation (Pearson coefficients) matrix between different yield and quality attributes of seventeen sweet potato genotypes cultivated in Bogura, Bangladesh during two consecutive growing seasons (2020–21 and 2021–22). Where, VL=vine length, ARL=average storage root length, ARD=average storage root diameter, MRN=marketable storage root number per plant, NMRN=average non-marketable storage root number per plant, NMRW=average non-marketable storage root weight per plant, MRY= Marketable storage root yield, BC=Beta-carotene content, VC= Vitamin-C, ST= starch, TS= total sugar, ACD= titratable acidity, DW=dry weight of storage root, TSS= total soluble solid.

Data for correlation analysis were gathered across two consecutive years, encompassing seventeen distinct varieties, as illustrated in Fig. 1. The findings revealed notable correlations (*r*= 0.21 to 0.84) among the studied traits, with statistical significance (*p* < 0.05).

### 3.3 Principal component and cluster analysis

**Fig. 2:**
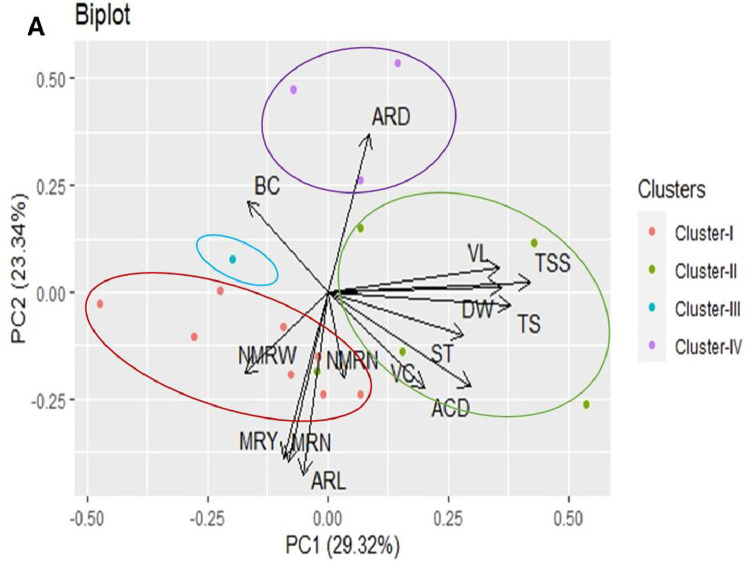
PCA biplot analysis of yield and quality traits and plotting of identified clusters of seventeen sweet potato genotypes based on their average performances of two consecutive growing seasons (2020–21 and 2021–22). Where, VL=vine length, ARL=average storage root length, ARD=average storage root diameter, MRN=marketable storage root number per plant, NMRN=average non-marketable storage root number per plant, NMRW=average non-marketable storage root weight per plant, MRY= Marketable storage root yield, BC=Beta-carotene content, VC= Vitamin-C, ST= starch, TS= total sugar, ACD= titratable acidity, DW=dry weight of storage root, TSS= total soluble solid.

The PCA biplot, which utilized the first two PCs among five significant PCs (Supplement Table 1) to represent both traits and genotypes, is presented in Fig. 2. To better grasp the relationships between genotypes and their associated attributes, the PCA biplot further delineated four clusters (depicted by circles in four different colors) (Fig. 2). Cluster I, indicated by the red-circle exhibited 8 genotypes. Cluster II, represented by the green circle, includes 5 genotypes. Cluster III comprises only 1 genotype, shown as the blue dot. Finally, Cluster IV (purple circle) encompasses 3 genotypes.

### 3.4 Factorial analysis

Sweet potato ideotypes were identified for two consecutive years by assessing the genetic correlation of each factor using the MGIDI index (Table 4). In the 2020–21 growing season, factor 1 correlated with VL, DW, TS, and TSS, while factor 2 was associated with MRN and MRY, and factor 3 with BC, VC, and ACD. Likewise, in the 2021–22 growing season, factor 1 was linked to VL, DW, and TS, factor 2 to TSS and MRN, and factor 3 to MRY, BC, VC, and ACD. The MGIDI index revealed a total desired genetic gain of 49.67 % and 136.37 % across the examined traits in two consecutive years, respectively. Broad-sense heritability (h2) ranged from 76.1 % to 99.8 % in the 2020–21 season and from 64.9% to 99.6% in the 2021–22 season. In the 2020–21 season, the average communality and uniqueness accounted for 78 % and 22 %, respectively. Similarly, in the 2021–22 season, the average communality and uniqueness contributed to 75.89 % and 24.11 %, respectively.

**Table 4:** Communalities (TU), uniqueness (TU), genetic gains (GG) and heritability (h2) obtained from factor analysis of MGIDI using two years’ performance of 17 sweet potato genotypes.

Traits	Sense	Year									
		2020–21					2021–22				
		Factors (FAs)	TC (%)	TU (%)	h^2^ (%)	GG (%)	Factors (FAs)	TC (%)	TU (%)	h^2^ (%)	GG (%)
VL	Positive	FA1	87	13	0.904	17.5	FA1	87	13	0.848	14.7
DW			89	11	0.991	28.8	FA1	94	6	0.984	27.9
TS			95	5	0.983	3.09	FA1	93	7	0.933	8.83
TSS			91	9	0.984	4.08	FA2	92	8	0.811	7.54
MRN		FA2	59	41	0.761	8.4	FA2	56	44	0.649	3.64
MRY			56	44	0.884	14.9	FA3	43	57	0.996	−19.2
BC		FA3	71	29	0.998	−29.3	FA3	62	38	0.991	75.4
VC			82	18	0.995	−19.9	FA3	76	24	0.932	1.86
ACD			72	28	0.995	22.1	FA3	80	20	0.974	15.7
			78^A^	22^A^		49.67^T^		75.89^A^	24.11^A^		136.37^T^

*A*=average value, *T*= total value, VL=vine length, ARL=average storage root length, ARD=average storage root diameter, MRN=marketable storage root number per plant, NMRN=average non-marketable storage root number per plant, NMRW=average non-marketable storage root weight per plant, MRY= Marketable storage root yield, BC=Beta-carotene content, VC= Vitamin-C, ST= starch, TS= total sugar, ACD= titratable acidity, DW=dry weight of storage root, TSS= total soluble solid

### 3.5 Multi trait genotype-ideotype index (MGIDI)

**Fig. 3:**
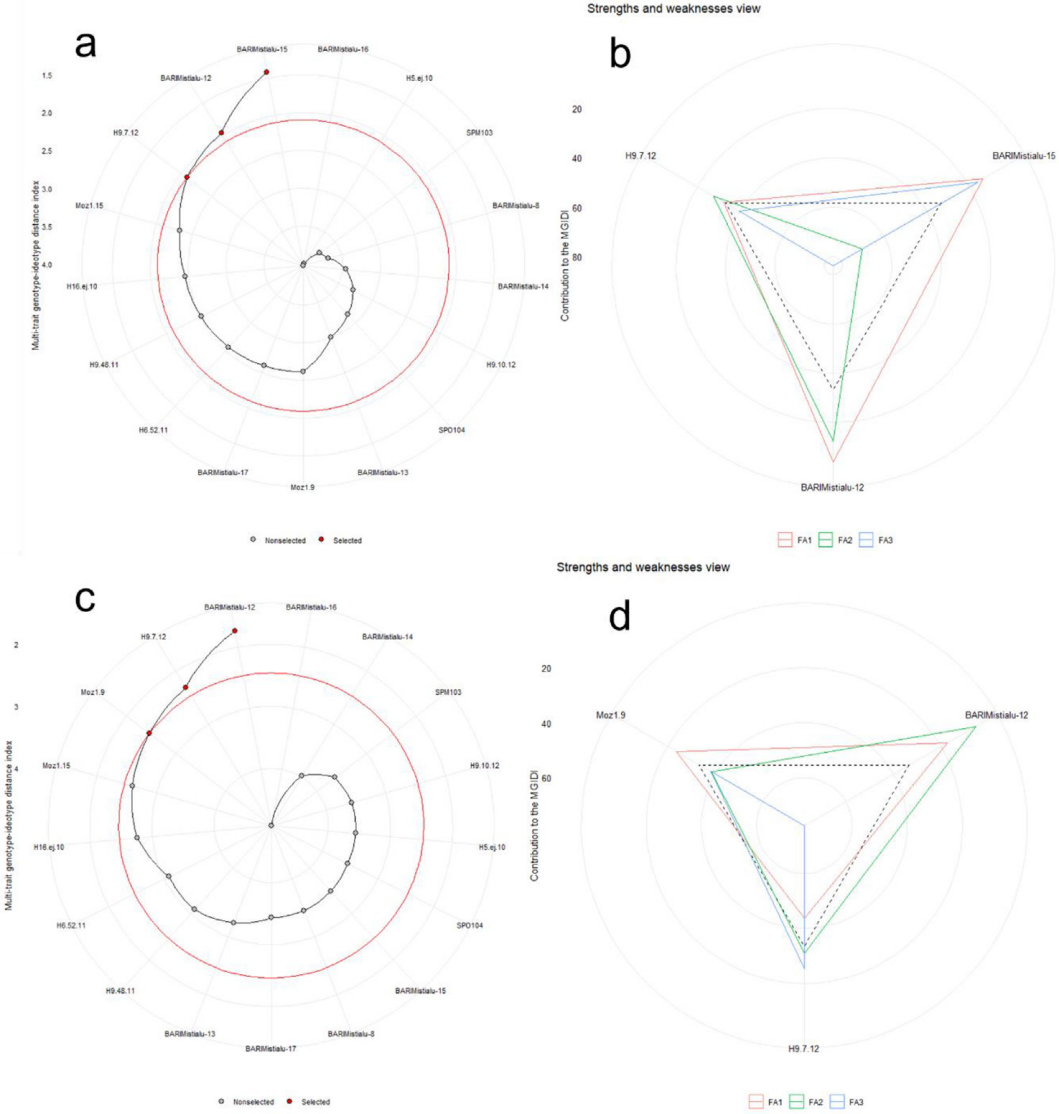
Ranking of sweet potato genotypes using MGIDI index based on multi trait performances (A for 2020–21 and C for 2021–22 growing season). The strengths and weaknesses of the selected genotypes for two consecutive growing seasons (b for 2020–21 and d for 2021–22 growing season). Where, FA1=factor1, FA2=factor2 and FA3=factor3.

**Fig. 4:**
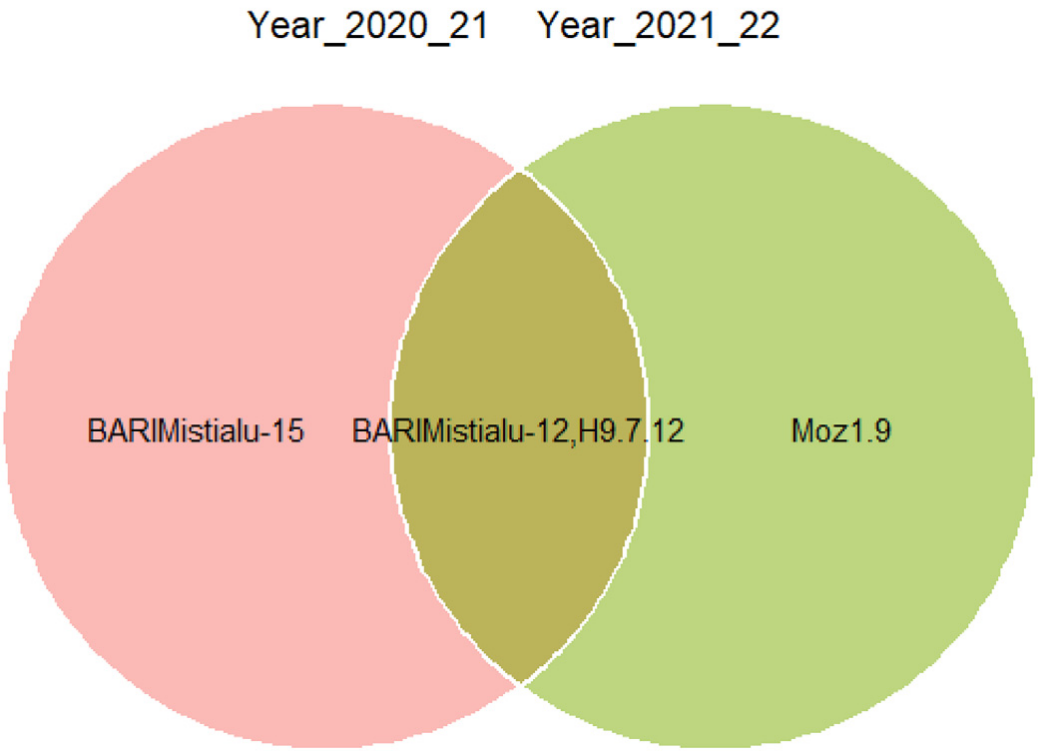
A Venn diagram of MGIDI index for two consecutive growing years.

The MGIDI index identified top-performing sweet potato genotypes based on yield and quality traits over two consecutive growing years (Fig. 3). In the 2020–21 season, BARI Mistialu-15 showed notable performance, followed by BARI Mistialu-12 and H9.7.12 (Fig. 3a). Conversely, in 2021–22, BARI Mistialu-12 emerged as the top performer, followed by H9.7.12 and Moz1.15 (Fig. 3c). Fig. 3b and d depict the strengths and weaknesses of these selected genotypes for each respective growing season. The Venn diagram revealed that genotypes BARI Mistialu-12 and H9.7.12 were found to be common across both years of study (Fig. 4).

## 4 Experimental Design, Materials and Methods

### 4.1 Experimental sites

The research spanned two consecutive growing seasons (2020–21 and 2021–22) in Bogura, Bangladesh, chosen for its favorable conditions for sweet potato cultivation and its alignment with Bangladeshʼs agroecology. The study site is situated at approximately 23.6850° N latitude and 90.3563° E longitude, with an elevation ranging from 10 m above sea level in the Coastal South to 105 m above sea level in the North.

### 4.2 Experimental materials and design

The experimental materials consisted of a range of sweet potato genotypes, including four imported genotypes (Moz.1.15, Moz.1.9, SPM-103, and SPO-104), six hybrids resulting from half diallel crosses (H9.7.12, H9.10.12, H6.52.11, H9.48.11, H5.ej.10, and H16.ej.10), and seven varieties released by BARI (BARI Mistialu-8, BARI Mistialu-12, BARI Mistialu-13, BARI Mistialu-14, BARI Mistialu-15, BARI Mistialu-16, and BARI Mistialu-17) obtained from TCRC, BARI, Bangladesh. All these genotypes mature approximately 130 days after vine planting. Supplementary Table 2 provides a detailed overview of the various sweet potato genotypes used. The experimental design employed was a randomized complete block design (RCBD) with three replications.

### 4.3 Management practices, harvesting and data collection

Planting of sweet potato vines/cuttings took place on November 1st in both growing years. Harvesting occurred 130 days after planting, with 10 randomly selected plants from each plot for every replication. Storage roots weighing 500 g each were collected from each genotype and analyzed at the Laboratory of Postharvest Technology Division, BARI. Standard management practices for sweet potato, as outlined by Alam et al., were followed [3]. Instructions for collecting data on various traits of sweet potatoes are outlined below.

### 4.4 Measurement of average vine length (VL) (cm)

Measurements were taken to determine the lengths of the vines of ten plants that were selected for the study. The average value was then computed for each plant.

### 4.5 Measurement of average storage root length (ARL) (cm)

The process by which the lengths of the mature storage roots of 10 randomly selected plants from each plot were measured and subsequently averaged.

### 4.6 Measurement of average storage root diameter (ARD) (cm)

To determine the mean of the storage root diameter, we computed the average circumference at the central section of fully developed storage roots from a randomly selected sample of ten plants.

### 4.7 Marketable storage root number per plant (MRN)

Ten plants were systematically chosen for sampling in each plot. The marketable storage roots of each sampled plant were then counted and totaled. The total number of marketable storage roots was divided by the number of sampled plants. It is important to note that marketable roots were defined as those that were undamaged, free of insect infestation, and weighed between 100 g and 500 g.

### 4.8 Number of non-marketable storage roots per plant (NMRN)

To determine the frequency of non-marketable storage roots in a given plot, a sorting and counting technique was employed once the plants reached maturity. This involved identifying and counting the non-marketable storage roots of 10 sampled plants from each plot and then dividing the resulting number by the total number of sampled plants. This precise approach allowed for the calculation of non-marketable storage roots per plant. Itʼs worth noting that any storage roots that were damaged, infected with insects or outside the weight range of 100–500 g were considered non-marketable.

### 4.9 Measurement of non-marketable storage root weight per plant (NMRW) (kg)

To determine the average weight of the non-marketable storage roots per plant, we computed the mean value of the fresh weight for the sampled plants. This involved taking into account only the non-marketable storage roots of the plants.

### 4.10 Measurement of marketable storage root yield (MRY) (t/ha)

Marketable storage root yield was obtained by dividing the average yield of marketable storage roots, which were obtained from a selective sample of plants, by the number of plants present in one hectare of land.

### 4.11 Measurement of the dry weight of storage roots (DW) (%)

Fresh sweet potato samples weighing 100 g were obtained from each plot and immediately sliced and weighed. The samples were then left undisturbed in a well-ventilated room for five days. Following this, they were subjected to a 24-h drying process in a laboratory oven at a constant temperature of 80 °C. The dry matter content (%) was determined using the formula established by [6], which involves calculating the weight of the sample (g) after drying and dividing it by the weight of the fresh sample (g), then multiplying the result by 100 to obtain the dry matter percentage.

### 4.12 **Analysis of beta-carotene content (BC) (**mg/100 g)

Modifications were made to Molla et al.’s [7] methodology to quantify beta-carotene levels. Freeze-dried sweet potato storage roots from Fisher Scientific Ltd. in the UK were mixed with acetone and petroleum ether. The resulting solution was then purified using metabolic potassium hydroxide (KOH), distilled water, and anhydrous sodium sulfate. The absorbance at 765 nm was measured using a UV–vis Double Beam Spectrophotometer against petroleum ether as a blank.

### 4.13 Measurements of starch content (STRC) (%)

Starch content was determined using the conventional method, with results expressed as a percentage of dry weight [8]. Homogenization of 5 g of material was done with hot 80 % ethanol, followed by several rounds of ethanol extraction and color removal with Anthrone reagent. Perchloric acid was used for the residual, and the mixture was centrifuged again, with the supernatant removed and the volume adjusted to 100 ml. Heating the mixture to boiling, 4 ml of Anthrone reagent was added and cooled quickly for UV-Spectrophotometer analysis of color intensity at 630 nm.

### 4.14 **Measurements of vitamin C content (VC) (mg/**100 g**)**

The determination of ascorbic acid (vitamin C) content was carried out following the procedure outlined by Ranganna [8]. This involved blending 10 g of samples for 2 min and homogenizing the mixture with 50 mL of 3 % cold meta-phosphoric (HPO_3_) acid. The homogenate was filtered to obtain clear supernatant samples for ascorbic acid assay. Aliquot samples were titrated with 2,6-dichlorophenolindophenol solution. The titer value was recorded for each sample. The 2,6-dichlorophenolindophenol solution was calibrated using an ascorbic acid standard solution. Results were expressed in mg/100 g.

### 4.15 Measurement of titratable acidity (ACD) (%)

The titration method was employed to conduct the analysis of titratable acidity (TA). A quantity of 10 g of disordered storage root samples was combined with 40 cubic centimeters of distilled water in a kitchen blender. Subsequently, the amalgamation underwent filtration with the utilization of cotton wool. A volume of 5 ml of the filtrate was subjected to titration with 0.1 Normal (N) Sodium Hydroxide (NaOH) until a pink endpoint was attained. To serve as an indicator, one to two drops of phenolphthalein (0.1 %) were incorporated into the process, with the pH level measured at 8.1. The outcomes were expressed in terms of the proportion of citric acid found in 100 g of fresh weight.

### 4.16 Measurement of total sugar (TS) (%)

In order to assess the total sugars present, sucrose, a non-reducing sugar, was converted to a reducing sugar through inversion. To do this, 2 g of citric acid were added to 20 ml of filtered extract and incubated at 60 °C until the inversion was complete. The resulting acid-hydrolyzed solution was then neutralized with NaOH. A 5 ml sample of the hydrolyzed solution was taken to calculate the total sugar in terms of invert sugar. Concentrations of sucrose and total sugars were determined using following formulas:

Sucrose(%)=(Totalinvertsugar−reducingsugar)×0.95

Totalsugar(%)=Totalsugar(%)+Sugar(%)

### 4.17 Measurement of total soluble solid (TSS) (%)

The total soluble solid in the juice created by mixing water and messed up the storage root was measured using a refractometer (ATAGO (Brix = 0 to 32 %)), and the findings were represented as a percentage of Brix.

### 4.18 Statistical analysis

The pooled ANOVA was conducted using the “agricolae” package in R statistical analysis software (Version 4.2.0) [9]. A Least Significant Difference test was employed to compare genotype and year means, with significance set at *p* < 0.05. Pearson correlation coefficients were calculated using the “metan” package at a significance level of *p* < 0.05. Principal Component Analysis (PCA) of trait mean data utilized the “ggplot2” and “ggfortify” packages. The MGIDI index and factor analysis for each year were carried out in R Studio using the “metan” package.

## Limitations

The study, despite its thorough examination of sweet potato genotypes and their traits, overlooks the consideration of adequate environmental factors or external variables that could impact genotype performance, potentially restricting the applicability of the results.

## Ethics Statement

All authors have read and follow the ethical requirements for publication in Data in Brief and our work meets these requirements. Our work does not involve studies with animals and humans.

## CRediT authorship contribution statement

**Zakaria Alam:** Conceptualization, Methodology, Investigation, Supervision, Visualization, Writing – original draft, Writing – review & editing. **Sanjida Akter:** Validation, Software. **Md. Anwar Hossain Khan:** Data curation. **Atiqur Rahman:** Validation. **Md Hasan Sofiur Rahman:** Conceptualization, Methodology.

## Data Availability

Ideotype based genotype selection in a multivariate dataset of sweet potato (Ipomoea batatas L.) (Original data) (Mendeley Data).

## Appendix Supplementary materials

Image, application 1
