# Supplementary material for: Ideotype based genotype selection in a multivariate dataset of sweet potato (Ipomoea batatas L.)
Source: Data Brief. 2024 Jun 1;55:110575. doi: 10.1016/j.dib.2024.110575 (PMC11214191; doi:10.1016/j.dib.2024.110575)
Supplement: Supplementary file 1 [file mmc1.docx]

Supplementary Table 1. Contributions of five principal components (PCs) for 14 yield and quality traits of 17 sweet potato genotypes evaluated in two growing years (2020-21 and 2021-22)

| Yield traits | Principal components | | | | |
| --- | --- | --- | --- | --- | --- |
|  | PC1 | PC2 | PC3 | PC4 | PC5 |
| VL | 0.38 | 0.06 | -0.28 | 0.28 | -0.29 |
| ARL | -0.06 | -0.46 | 0.00 | -0.12 | 0.06 |
| ARD | 0.09 | 0.40 | -0.33 | 0.19 | 0.07 |
| MRN | -0.09 | -0.43 | -0.45 | -0.03 | 0.12 |
| NMRN | 0.04 | -0.21 | 0.14 | 0.58 | 0.40 |
| NMRW | -0.18 | -0.20 | 0.23 | 0.50 | -0.07 |
| MRY | -0.10 | -0.42 | -0.46 | -0.05 | 0.09 |
| Quality traits | | | | | |
| DW | 0.39 | 0.01 | -0.29 | 0.35 | -0.09 |
| BC | -0.18 | 0.23 | 0.07 | 0.17 | 0.58 |
| VC | 0.21 | -0.24 | 0.33 | -0.01 | -0.29 |
| ST | 0.30 | -0.11 | 0.16 | -0.24 | 0.38 |
| TS | 0.41 | -0.03 | 0.05 | -0.13 | 0.30 |
| TSS | 0.45 | 0.03 | -0.05 | -0.19 | 0.21 |
| ACD | 0.32 | -0.24 | 0.31 | 0.12 | -0.11 |
| %Explained | 29.32 | 23.34 | 10.84 | 10.12 | 7.17 |
| % Cumulative explained | 29.32 | 52.66 | 63.50 | 73.62 | 80.79 |
| Eigenvalue | 4.11 | 3.27 | 1.52 | 1.42 | 1.00 |

Supplementary Table 2: Descriptions of seventeen sweet potato genotypes grown in Bogura, Bangladesh during two growing seasons (2020-21 and 2021-22)

| Genotype | Genotype ID | Origin | Altitude (m) | Year of release | Potential yield (t/ha) |
| --- | --- | --- | --- | --- | --- |
| Moz.1.15 |  | CIP, Mozambique | 345 | NR | NS |
| Moz.1.9 |  | CIP, Mozambique | 345 | NR | NS |
| SPM-103 |  | Japan | 438 | NR | NS |
| SPO-104 |  | Japan | 438 | NR | NS |
| H9.7.12 |  | H (BD) | 105 | NR | NS |
| H9.10.12 |  | H (BD) | 105 | NR | NS |
| H6.52.11 |  | H (BD) | 105 | NR | NS |
| H9.48.11 |  | H (BD) | 105 | NR | NS |
| H5.ej.10 |  | H (BD) | 105 | NR | NS |
| H16.ej.10 |  | H (BD) | 105 | NR | NS |
| BARI Mistialu-12 |  | CIP, Peru | 1555 | 2013 | 35-40 |
| BARI Mistialu-16 |  | H (BD) | 105 | 2018 | 35-40 |
| BARI Mistialu-17 |  | Indonesia | 367 | 2021 | 30-35 |
| BARI Mistialu-8 |  | CIP, Peru | 1555 | 2008 | 30-35 |
| BARI Mistialu-13 |  | CIP, Peru | 1555 | 2013 | 30-35 |
| BARI Mistialu-14 |  | H (BD) | 105 | 2013 | 30-35 |
| BARI Mistialu-15 |  | CIP, Peru | 1555 | 2017 | 35-40 |

H(BD)= hybridization in Bangladesh, CIP= International Potato Centre, NR= not released yet, NS= not stable yet

Supplementary Table 3. Eigenvalues and contribution (%) of principal components (PCs) for yield and quality traits (VL, MRN, MRY, DW, BC, VC, TS, TSS and ACD) of 17 sweet potato genotypes obtained from factor analysis of MGIDI index during 2020-21 and 2021-22

| Year | | | | | | |
| --- | --- | --- | --- | --- | --- | --- |
| 2020-21 | | | | 2021-22 | | |
| PC | Eigenvalues | Variance (%) | Cumulative variance (%) | Eigenvalues | Variance (%) | Cumulative variance (%) |
| PC1 | 3.65 | 40.6 | 40.6 | 3.64 | 40.4 | 40.4 |
| PC2 | 2.17 | 24.1 | 64.7 | 2.07 | 23 | 63.4 |
| PC3 | 1.2 | 13.4 | 78 | 1.12 | 12.4 | 75.8 |
| PC4 | 0.83 | 9.2 | 87.2 | 0.89 | 9.93 | 85.8 |
| PC5 | 0.65 | 7.22 | 94.5 | 0.7 | 7.8 | 93.6 |
| PC6 | 0.37 | 4.17 | 98.6 | 0.38 | 4.21 | 97.8 |
| PC7 | 0.09 | 0.96 | 99.6 | 0.12 | 1.32 | 99.1 |
| PC8 | 0.02 | 0.27 | 99.8 | 0.07 | 0.81 | 99.9 |
| PC9 | 0.01 | 0.15 | 100 | 0.01 | 0.11 | 100 |

Supplementary Table 4. Factorial loadings (FA) based on the factor analysis (PC eigenvalue >1) of MGIDI index of two growing years (2020-21 and 2021-22)

| Year | | | | | | |
| --- | --- | --- | --- | --- | --- | --- |
| 2020-21 | | | | 2021-22 | | |
| Traits | FA1 | FA2 | FA3 | FA1 | FA2 | FA3 |
| VL | 0.93 | 0 | 0.09 | 0.92 | 0.1 | 0.11 |
| MRN | -0.06 | 0.94 | 0.09 | 0.03 | -0.97 | 0.01 |
| MRY | -0.01 | 0.98 | 0 | -0.1 | -0.95 | 0.11 |
| DW | 0.94 | 0.13 | 0.09 | 0.95 | 0.1 | 0.14 |
| BC | -0.14 | -0.33 | -0.68 | -0.01 | 0.24 | -0.71 |
| VC | 0 | -0.03 | 0.75 | 0.23 | 0.04 | 0.61 |
| TS | 0.58 | -0.34 | 0.51 | 0.53 | -0.12 | 0.57 |
| TSS | 0.69 | -0.37 | 0.45 | 0.76 | -0.09 | 0.41 |
| ACD | 0.34 | -0.04 | 0.78 | 0.21 | 0.07 | 0.86 |

Supplementary Table 5. Rescaled BLUP (best linear unbiased prediction) values for 9 yield and quality traits measured in 17 sweet potato genotypes (Growing season 2020-21)

| Genotype | VL | MRN | MRY | DW | BC | VC | TS | TSS | ACD |
| --- | --- | --- | --- | --- | --- | --- | --- | --- | --- |
| G1 | 107 | 7.29 | 46.2 | 23.1 | 8.41 | 5.49 | 11 | 10.7 | 0.129 |
| G2 | 137 | 5.27 | 32.6 | 32.8 | 8.41 | 23.2 | 12.2 | 12.4 | 0.326 |
| G3 | 102 | 5.72 | 30.3 | 19.4 | 9.04 | 11.2 | 10.3 | 9.3 | 0.134 |
| G4 | 114 | 5.41 | 35.4 | 21.2 | 13.2 | 4.93 | 9.37 | 9.17 | 0.133 |
| G5 | 133 | 7.4 | 49.3 | 31.9 | 13.8 | 8.75 | 9.2 | 8.99 | 0.134 |
| G6 | 102 | 6.95 | 42 | 19.8 | 20.8 | 4.87 | 8.85 | 8.84 | 0.126 |
| G7 | 107 | 6.35 | 36.1 | 23.1 | 12.9 | 5.27 | 11 | 10.9 | 0.202 |
| G8 | 107 | 6.5 | 39.8 | 22.4 | 6.7 | 5.13 | 11.4 | 10.4 | 0.206 |
| G9 | 103 | 5.36 | 30.9 | 23.2 | 49.1 | 4.53 | 9.69 | 9.35 | 0.126 |
| G10 | 131 | 5.26 | 30.8 | 28.1 | 9.46 | 3.99 | 10.5 | 10.8 | 0.134 |
| G11 | 142 | 6.97 | 43.8 | 35.9 | 16.9 | 4.29 | 10.6 | 10.4 | 0.138 |
| G12 | 104 | 6.05 | 40 | 19.9 | 21.1 | 4.33 | 5.7 | 6.71 | 0.135 |
| G13 | 112 | 6.73 | 36 | 26 | 16 | 11.8 | 10.2 | 8.81 | 0.213 |
| G14 | 124 | 4.83 | 26.3 | 28.2 | 34.9 | 3.3 | 11.3 | 11.3 | 0.0811 |
| G15 | 117 | 5.26 | 27.7 | 25.5 | 18.8 | 4.61 | 11 | 10.7 | 0.177 |
| G16 | 122 | 4.82 | 28.8 | 23.4 | 16.7 | 4.8 | 9.6 | 9.26 | 0.0761 |
| G17 | 149 | 5.56 | 32.4 | 31.4 | 4.28 | 2.97 | 11.8 | 11.8 | 0.332 |
| Significance | * | | | | | | | | |

*Significant (p<0.05) genotype effect

Supplementary Table 6. Rescaled BLUP (best linear unbiased prediction) values for 9 yield and quality traits measured in 17 sweet potato genotypes (Growing season 2021-22)

| Genotype | VL | MRN | MRY | DW | BC | VC | TS | TSS | ACD |
| --- | --- | --- | --- | --- | --- | --- | --- | --- | --- |
| G1 | 114 | 6.36 | 39.5 | 23.9 | 9.38 | 5.24 | 11.4 | 11.2 | 0.136 |
| G2 | 145 | 5.31 | 32.8 | 34.1 | 9.58 | 24.4 | 12.2 | 13.1 | 0.294 |
| G3 | 114 | 5.01 | 31.7 | 22.1 | 9.97 | 11.5 | 10.7 | 9.78 | 0.134 |
| G4 | 110 | 5.41 | 31 | 22.8 | 13 | 5.18 | 9.9 | 9.53 | 0.134 |
| G5 | 136 | 5.49 | 32.8 | 31.8 | 14 | 9.54 | 9.61 | 9.67 | 0.134 |
| G6 | 111 | 5.53 | 35.1 | 20.1 | 22.2 | 5.3 | 9.43 | 9.27 | 0.127 |
| G7 | 109 | 5.52 | 34.1 | 22.6 | 13.7 | 5.57 | 11.3 | 11.2 | 0.194 |
| G8 | 109 | 5.71 | 40.1 | 22.7 | 6.69 | 5.69 | 11.9 | 10.8 | 0.205 |
| G9 | 111 | 4.96 | 32 | 23.5 | 48.6 | 5.34 | 10.4 | 10.1 | 0.133 |
| G10 | 134 | 4.86 | 29.4 | 28.9 | 10.6 | 5.04 | 10.6 | 11.1 | 0.131 |
| G11 | 149 | 6.2 | 37.4 | 36.1 | 17.8 | 4.83 | 10.5 | 11.3 | 0.142 |
| G12 | 108 | 4.79 | 32.5 | 19.4 | 21.7 | 4.51 | 6.45 | 6.73 | 0.136 |
| G13 | 117 | 5.22 | 31.9 | 26.5 | 17.4 | 12 | 11 | 8.5 | 0.215 |
| G14 | 130 | 4.32 | 28.1 | 30 | 34.8 | 4.34 | 11.4 | 11.4 | 0.0828 |
| G15 | 123 | 4.79 | 28.1 | 28 | 19.7 | 5.29 | 10.9 | 10.7 | 0.176 |
| G16 | 119 | 4.48 | 27.2 | 24.8 | 17.7 | 5.98 | 9.4 | 9.76 | 0.0812 |
| G17 | 137 | 4.17 | 28.8 | 32.5 | 3.75 | 4.92 | 12.2 | 12.3 | 0.329 |
| Significance | * | | | | | | | | |

*Significant (p<0.05) genotype effect
